# A risk measurement tool for targeted HIV prevention measures amongst young pregnant and lactating women in South Africa

**DOI:** 10.1186/s12889-022-13625-8

**Published:** 2022-06-30

**Authors:** Trisha Ramraj, Nada Abdelatif, Witness Chirinda, Fareed Abdullah, Gurpreet Kindra, Ameena Goga

**Affiliations:** 1 Health Systems Research Unit, South African Medical Research Council, 491 Peter Mokaba Ridge, Durban, 4001 South Africa; 2 Present Address: HIV and other Infectious Diseases Research Unit, South African Medical Research Council, 491 Peter Mokaba Ridge, Durban, 4001 South Africa; 3 Biostatistics Research Unit, South African Medical Research Council, 491 Peter Mokaba Ridge, Durban, 4001 South Africa; 4 Office of AIDS and TB Research, South African Medical Research Council, 1 Soutpansberg Road, Pretoria, 0001 South Africa; 5 Division of Infectious Diseases, University of Pretoria, Pretoria, SA South Africa; 6 US Centers for Disease Control and Prevention, Division of Global HIV and Tuberculosis, Center for Global Health, Pretoria, SA South Africa; 7 Department of Paediatrics and Child Health, University of Pretoria, Pretoria, SA South Africa

**Keywords:** HIV, Risk score, Young women, Pregnant, Lactating, South Africa

## Abstract

**Background:**

We aimed to develop and validate a tool to identify which pregnant/lactating young South African women (≤ 24 years) are at risk of HIV infection.

**Methods:**

Data from three national South African Prevention of Mother-to-Child Transmission (PMTCT) evaluations were used to internally validate three HIV acquisition risk models for young postpartum women. We used univariate and multivariable logistic regression analysis to determine which risk factors were significant. Model coefficients were rounded and stratified into risk groups and the area under the receiver operating curve (AUROC) was computed. Models were developed to determine which risk factors provided the most predictive accuracy whilst remining clinically meaningful.

**Results:**

Data from 9 456 adult and 4 658 young pregnant and lactating women were included in the development and validation data sets, respectively. The optimal model included the following risk factors: age (20–24 years old), informal house structure, two or more pregnancies, mothers who had knowledge of when they received their last HIV test result, no knowledge of the infant’s father’s HIV status, no knowledge of breastfeeding as a mode of MTCT and knowledge of PMTCT programme. The mean AUROC was 0.71 and 0.72 in the development and validation datasets respectively. The optimum cut off score was ≥ 27, having 84% sensitivity, 44% specificity, and identifying 44% of high-risk women eligible for PrEP.

**Conclusion:**

The optimal model to be used as a possible risk scoring tool to allow for early identification of those pregnant/lactating women most at-risk of HIV acquisition included both statistically as well as clinically meaningful risk factors. A field-based study is needed to test and validate the effectiveness of this targeted approach.

## Background

The progress made towards reducing AIDS-related deaths has not been matched with equal progress in reducing new HIV infections [1]. Vulnerable young women continue to account for a disproportionate number of new infections [1]. In 2018, 210 000 young women aged 15–24 years were newly infected with HIV in sub-Saharan Africa compared to 87 000 young men of the same age [2]. The risk of HIV acquisition is increased during pregnancy and postpartum; thus women from regions with high HIV prevalence and high fertility rates have a sustained cumulative risk of HIV acquisition through a substantial portion of their life course [3]. In a systematic review of 19 cohorts and 22 803 person-years, Drake et al. found that the pooled HIV incidence was 4.7 and 2.9 per 100 person-years in pregnant and postpartum women respectively [3]. Furthermore, the pooled cumulative HIV incidence was significantly higher among African (3.6%) compared to non-African (0.3%) countries [3]. Incident HIV infections during pregnancy or postpartum lead to an increased risk of mother-to-child transmission (MTCT) of HIV [3, 4].

An intensified shift towards targeted uptake of a combination of prevention interventions by at risk individuals such as pregnant/postpartum women during periods of increased risk is needed to reduce new infections [5]. Combination prevention packages include biomedical (pre-exposure prophylaxis (PrEP), post-exposure prophylaxis, condom use), behavioural (HIV testing and risk reduction counselling, cash incentives) and structural interventions (community awareness, advocacy, promoting disclosure and reducing stigma) to reduce new infections [6, 7]. To maximise impact, be cost effective, and use resources optimally, combination prevention packages must be targeted at specific locations and at specific key and vulnerable populations [6, 7]. The World Health Organization recommended that people at substantial risk of HIV i.e. HIV incidence of 3 per 100 person-years or higher in the absence of PrEP – should be offered PrEP [8]. The South African guidelines have been amended to include this recommendation thus allowing a wider range of populations to benefit from PrEP [9]. Adolescent girls and young women are considered to be a population at substantial risk of HIV and are thus potential candidates for PrEP. Risk measurement tools have an important role to play in identifying which subgroups are most at risk of HIV acquisition and subsequently identify the optimal target population for HIV prevention interventions such as PrEP [10].

Risk scoring tools have been designed to identify individuals at increased risk of HIV acquisition. As the implementation of PrEP programmes expands, the ability to effectively prioritize PrEP delivery through the use of risk scoring tools, especially in resource constrained settings, allow for targeted provision of these services resulting in a potential reduction in transmission [11]. Considering that young women account for the highest proportion of annual new infections[12] and that the risk of incident HIV infections during pregnancy are high, the antenatal and early postpartum period provides an opportunity to offer this population HIV-1 prevention strategies such as early ART initiation and PrEP [3]. Previous studies have developed risk scoring tools for men who have sex with men [13, 14], HIV-serodiscordant couples [15, 16], women [17] and pregnant women [5]. A study among pregnant and postpartum women in Kenya found that a composite risk score including behavioral (number of lifetime sexual partners, male partner HIV status) and clinical characteristics (syphilis, bacterial vaginosis and vaginal candidiasis) had good predictive ability to identify pregnant and postpartum women most likely to acquire HIV [5]. Using data from three national South African Prevention of MTCT (PMTCT) programme evaluations conducted between 2010–2013, this analysis aimed to develop a risk measurement tool to identify which pregnant/lactating young women (≤ 24 years) are at risk of HIV infection. Targeting this high risk subgroup of young women who have high fertility (age specific fertility rate of 71 and 133 among women aged 15–19 and 20–24 years respectively) and HIV incidence rates (2.0% among 15–24 year old women) could maximize the impact of HIV prevention interventions and outweigh any potential risk of PrEP use during pregnancy and lactation, thereby optimally controlling new HIV infections amongst young women during pregnancy and lactation [18, 19].

## Methods

### Study design, setting and population

We combined data from three nationally-representative, cross sectional facility-based surveys conducted in 2010 (June-December 2010), 2011–12 (August 2011-March 2012), and 2012–13 (October 2012-May 2013). The main outcome of the primary surveys was to estimate early (4–8 weeks postpartum) MTCT. A full description of the sampling, methodology and main findings of the primary surveys is reported elsewhere [20, 21]. In each survey, probability proportional to population size methodology was used to randomly select 580 public health facilities (34–79 facilities per province) across all nine provinces of South Africa. The main eligibility criteria were consistent across the three surveys and included infants aged 4–8 completed weeks and their caregivers visiting public primary healthcare clinics (PHC) or community health centres (CHCs) for their 6-week vaccine (as per Expanded Programme on Immunization schedule) on the day of the visit.

### Study procedures

Trained study nurses conducted face-to-face interviews with eligible mother-infant pairs. Data were collected electronically on mobile phones and uploaded into a web-based interface. Self-reported data on maternal age, socio-demographics, antenatal and postnatal care, infant health and feeding practices, knowledge about MTCT, HIV testing, maternal and partner HIV status, PMTCT care during pregnancy and delivery were collected.

### Ethical considerations

The protocols, informed consent forms, and questionnaires from the 2010, 2011–12, and 2012–13 surveys were approved by the South African Medical Research Council’s ethics committee and relevant provincial research ethics committees. Approval was obtained from the U.S. Centers for Disease Control and Prevention’s (Atlanta, GA) Center for Global Health’s Associate Director for Science.

### Risk factors

The analysis considered demographic and socioeconomic factors such as age (< 20 and 20–24 years), ethnicity, marital status (single/divorced/widowed and married/cohabiting), education level of mother, income (own employment, from family or grants), knowing how many weeks pregnant the mother was before she received her last HIV test result, knowledge of father’s HIV status, type of dwelling, type of toilet and fuel used for cooking, the number of pregnancies the mother has had, and the number of children the mother has had. Knowledge of modes of MTCT was measured by asking mothers two questions “Can a baby get HIV from the mother if the mother is infected with HIV?” and if yes to this question the mother was asked “How?” (multiple response question with the options breastfeeding, during pregnancy, during childbirth, other, don’t know). Knowledge of PMTCT programme was measured from a single question “Have you ever heard of the PMTCT or Prevention of mother-to-child transmission programme?” Pregnancy planning was measured from a single question referring to the most recent pregnancy – “Was this pregnancy planned?”. These questions were closed-ended with pre-specified options asked by an interviewer; however, an “other” option was included to allow women to specify a response not included in the response options of the questionnaire.

### Dependent variable

For the dependent variable, two questions captured self-reported maternal HIV status: 1. What was your HIV result prior to the last pregnancy? (This referred to any test done prior to the last pregnancy); 2. What was your latest HIV result? (This referred to a test done during this last pregnancy or delivery). Women who reported being HIV positive from the first question were not asked the second the question. All factors were parametrized as categorical variables and only non-missing observations were used in the analyses.

### Statistical analysis

A split-sample method was used to develop a risk equation and scoring system, with participants allocated randomly to either the development data set (67%) or internal validation data set (33%). All analyses were carried out in Stata 15 (StataCorp., 2017).

### Risk score development and validation

The development data set was used to develop the risk model. Three risk models were developed and assessed to determine which risk factors provided the most predictive accuracy whilst remaining clinically meaningful. Model I included, in a multivariable logistic regression model, predictors that were statistically significantly associated with self-reported HIV positive or negative status at *p*-value < 0.10 in the univariate logistic regression. This *p*-value was used to gauge which predictors could potentially be statistically significant in the multivariable analyses. To select the best fitting model, predictors were added both individually and in combinations to assess their importance and contribution. Model II is the same as Model I but with the removal of ethnicity, since the study population was predominantly of one ethnicity, resulting in this variable adding more weight to the risk scoring system than is justified. Clinically meaningful predictors i.e., risk factors that can be easily identified in routine clinical settings were used in Model III. To determine which of these predictors should be included in the final multivariable logistic regression model, bootstrap regression model with replacement was generated with 1 000 random samples. The predictors that occurred in more than 50% of the bootstrap models were included in the final regression model.

A weighted scoring system was then created by rounding the model coefficients of the three-final multivariable logistic models to the nearest integer and scaling the coefficients by multiplying by 10 to provide easier use in the calculations. The sum of the values for each predictor was then used to estimate participant-specific probabilities of HIV positivity and then cut-off points for degrees of risk based on probability distribution. Internal validation of the risk scoring tool was performed on the remaining 33% of participants. The area under the receiver-operating curve (AUROC) was used to assess the model correctness and their discriminative power of the scoring algorithm. Validation measures such as sensitivity and specificity and AUROC were computed as discrimination statistics and used to guide the final recommendation. To check the robustness of the risk scoring models, the development and validation data sets were randomly reselected five times.

## Results

### Participant demographic and social profile

Table 1 shows the characteristics of the young pregnant and lactating women (< 20 and 20–24 years old) in the study. Mothers < 20 years old accounted for 34% and those 20–24 years old were 66% of the study population used in this analysis. The majority of mothers were Black African (90%), single (88%), had an educational level of high school or more (88%), and 71% considered their pregnancies as unplanned. Most of the mothers had one child (68%) and/or one pregnancy (64%). Socioeconomic factors of the mothers in the study showed that 77% lived in a brick or cement house structure, 51% used pit latrine or portable toilets, whereas 49% used toilets that flush; and 91% used electricity or gas or paraffin to cook. Most of the mothers received their income from family members (90%) and 9% from own employment; only 11% received social grants. The majority of mothers knew the duration of their pregnancy when they received their last HIV test result (87%); however, only 51% knew the HIV status of the child’s father. Ninety percent (90%) of mothers had knowledge of breastfeeding as a mode of MTCT and 78% had knowledge of the PMTCT programme.

**Table 1:** Demographic and social profile of the development and validation data sets from the 2010, 2011–2012, and 2012–2013 prevention of mother-to-child HIV transmission surveys, South Africa

		**Development Data Set (*N* = 9456)**	**Validation Data Set (*N* = 4658)**	**Total Data Set (*N* = 4114)**
**Risk factor**		**N (%)**		
Age (years)	< 20	3192 (33.76)	1670 (35.85)	4862 (34.45)
	20–24	6264 (66.24)	2988 (64.15)	9252 (65.55)
Ethnicity	Black	8,498 (89.87)	4213 (90.45)	12,711 (90.06)
	Non-black	958 (10.13)	445 (9.55)	1403 (9.94)
Marital status of mother	Single/divorced/widowed	8296 (87.75)	4022 (86.35)	12,318 (87.29)
	Married	1158 (12.25)	636 (13.65)	1794 (12.71)
Educational level of mother	Primary or less	1174 (12.42)	607 (13.04)	1781 (12.63)
	High school or more	8276 (87.58)	4049 (86.96)	12,325 (87.37)
Type of house	Brick/cement	7249 (76.68)	3583 (76.94)	10,832 (76.76)
	Informal	2205 (23.32)	1074 (23.06)	3279 (23.24)
Type of toilet	Flush	4608 (48.74)	2296 (49.30)	6904 (48.93)
	Pit-latrine/Portable/other	4846 (51.26)	2361 (50.70)	7207 (51.07)
Fuel used for cooking	Electricity/gas/paraffin	8623 (91.21)	4250 (91.26)	12,873 (91.23)
	Wood/coal/other	831 (8.79)	407 (8.74)	1238 (8.77)
Income from own employment	No	8604 (91.01)	4277 (91.84)	12,881 (91.28)
	Yes	850 (8.99)	380 (8.16)	1230 (8.72)
Income from family	No	933 (9.87)	452 (9.71)	1385 (9.82)
	Yes	8521 (90.13)	4205 (90.29)	12,726 (90.18)
Income from grants	No	8405 (88.9)	4133 (88.75)	12,538 (88.85)
	Yes	1049 (11.1)	524 (11.25)	1573 (11.15)
Planned pregnancy	No	6390 (71.14)	3089 (69.82)	9479 (70.71)
	Yes	2592 (28.86)	1335 (30.18)	3927 (29.29)
Knowledge of weeks pregnant when received last HIV test result	No	613 (13.09)	298 (12.84)	911 (13.01)
	Yes	4069 (86.91)	2023 (87.16)	6092 (86.99)
Knowledge of HIV status of child’s father	No	2934 (51.02)	1438 (50.71)	4372 (50.91)
	Yes	2817 (48.98)	1398 (49.29)	4215 (49.09)
Number of pregnancies	1	5782 (64.19)	2867 (64.64)	8649 (64.34)
	> = 2	3226 (35.81)	1568 (35.36)	4794 (35.66)
Number of children	1	6118 (67.91)	3037 (68.48)	9155 (68.1)
	> = 2	2891 (32.09)	1398 (31.52)	4289 (31.9)
Knowledge of breastfeeding as a mode of MTCT	No	867 (10.25)	422 (10.12)	1289 (10.21)
	Yes	7590 (89.75)	3747 (89.88)	11,337 (89.79)
Knowledge of PMTCT programme	No	2102 (22.23)	1058 (22.72)	3160 (22.39)
	Yes	7352 (77.77)	3599 (77.28)	10,951 (77.61)

*MTCT* mother-to-child transmission, *PMTCT* prevention of mother-to-child transmission

### Development of risk scoring algorithm

The risk factors of HIV acquisition in young mothers from Model I were age (20–24 years old), informal house structure, having a flush toilet, two or more pregnancies, unplanned pregnancy, mothers who had knowledge of when they received their last HIV test result, no knowledge of the infant’s father’s HIV status, ethnicity, no knowledge of breastfeeding as a mode of MTCT, and knowledge of PMTCT programme (Table 2). The analysis was repeated using the internal validation data set; the results showed some similar findings, except for type of house, type of toilet, number of pregnancies and planned pregnancy, which were not significant. Higher scores were assigned to ethnicity and whether the mother had knowledge about PMTCT in the validation data set than the development data set.

**Table 2:** Final multivariable logistic regression of young mother’s HIV infection using the development and validation data sets for Model One from the 2010, 2011–2012, and 2012–2013 prevention of mother-to-child transmission surveys, South Africa

		**Development Data Set (*N* = 4230)**				**Validation Data Set (*N* = 2100)**				
**Risk factor**		**Coefficient**	**Odds Ratio (95% CI)**	***P*-value**	Score (coefficient*10)	**Coefficient**	**Odds Ratio (95% CI)**	***P*-value**		Score (coefficient*10)
Age of mother (years)	< 20	0	1	-	**0**	0	1		-	**0**
	20–24	0.81	2.26 (1.62–3.14)	< 0.001	**8**	0.75	2.11 (1.38–3.23)		0.001	**7**
Planned pregnancy	No	0.52	1.68 (1.24–2.27)	0.001	**5**					
	Yes	0	1	-	**0**					
Knowledge of weeks pregnant when received last HIV test result	No	0	1	-	**0**	0	1		-	**0**
	Yes	0.83	2.30 (1.39–3.82)	0.001	**8**	0.68	1.98 (1.01–3.85)		0.046	**7**
Knowledge of HIV status of child’s father	No	0.82	2.26 (1.74–2.93)	< 0.001	**8**	0.71	2.04 (1.40–2.96)		< 0.001	**7**
	Yes	0	1	-	**0**	0	1		-	**0**
Ethnicity	Black	1.61	5.03 (2.32–10.89)	< 0.001	**16**	2.56	12.94 (1.79–93.59)		0.011	**26**
	Non-black	0	1	-	**0**	0	1		-	**0**
Knowledge of breastfeeding as a mode of MTCT	No	0.82	2.28 (1.60–3.25)	< 0.001	**8**	0.77	2.17 (1.29–3.64)		0.003	**8**
	Yes	0	1	-	**0**	0	1		-	**0**
Knowledge of PMTCT programme	No	0	1	-	**0**	0	1		-	**0**
	Yes	0.67	1.95 (1.32–2.88)	0.001	**7**	1.00	2.71 (1.39–5.27)		0.003	**10**
Number of pregnancies	1	0	1	-	**0**					
	> = 2	0.49	1.64 (1.26–2.12)	< 0.001	**5**					
Type of house	Brick/cement	-	-	-	-					
	Informal	0.35	1.42 (1.07–1.89)	0.016	**4**					
Type of toilet	Flush	0.28	1.32 (1.02–1.72)	0.035	**3**					
	Pit-latrine/Portable/other	0	1	-	**0**					

*CI* Confidence interval, *MTCT* mother-to-child transmission, *PMTCT* prevention of mother-to-child transmission

The *p*-value was set at *p*-value < .05

In Model II, ethnicity was removed from the development and validation models. Eight risk factors were significant in the development data set (Table 3), and only toilet type was excluded from the Model II in comparison to Model I. Type of house and planned pregnancy were not significant risk factors in the development and validation data sets respectively. The scores differed slightly between the development and validation data sets for Model II, except for knowledge about the number of weeks pregnant when received last HIV test result, which was given a higher score of 12 in the validation data set compared to a score of 7 in the development data set.

**Table 3:** Final multivariable logistic regression of young mother’s HIV infection using the development and validation data sets for Model Two from the 2010, 2011–2012, and 2012–2013 prevention of mother-to-child transmission surveys, South Africa

		**Development Data Set (*N* = 4222)**				**Validation Data Set (*N* = 2110)**			
**Risk factor**		**Coefficient**	**Odds Ratio (95% CI)**	***P*-value**	Score (coefficient*10)	**Coefficient**	**Odds Ratio (95% CI)**	***P*-value**	Score (coefficient*10)
Age of mother (years)	< 20	0	1	-	**0**	0	1	-	**0**
	20–24	0.74	2.10 (1.50–2.94)	< 0.001	**7**	0.85	2.35 (1.54–3.58)	< 0.001	**9**
Planned pregnancy	No	0.67	1.95 (1.41–2.69)		**7**				
	Yes	0	1	-	**0**				
Knowledge of weeks pregnant when received last HIV test result	No	0	1	-	**0**	0	1	-	**0**
	Yes	0.65	1.92 (1.18–3.11)	0.008	**7**	1.16	3.18 (1.52–6.60)	0.002	**12**
Knowledge of HIV status of child’s father	No	0.81	2.25 (1.71–2.96)	< 0.001	**8**	0.75	2.12 (1.52–2.96)	< 0.001	**8**
	Yes	0	1	-	**0**	0	1	-	**0**
Knowledge of breastfeeding as a mode of MTCT	No	0.77	2.16 (1.49–3.11)	< 0.001	**8**	0.78	2.19	0.001	**8**
	Yes	0	1	-	**0**	0	1	-	**0**
Knowledge of PMTCT programme	No	0	1	-	**0**	0	1	-	**0**
	Yes	0.84	2.31 (1.53–3.49)	< 0.001	**8**	0.98	2.65 (1.53–4.60)	0.001	**10**
Number of pregnancies	1	0	1	-	**0**	0	1	-	**0**
	> = 2	0.45	1.57 (1.19–2.06)	0.001	**4**	0.41	1.51 (1.07–2.12)	0.019	**4**
Type of house	Brick/cement					0	1	-	**0**
	Informal					0.40	1.50 (1.04–2.14)	0.028	**4**

*CI* Confidence interval, *MTCT*, mother-to-child transmission, *PMTCT* prevention of mother-to-child transmission

The *p*-value was set at *p*-value < .05

For Model III, clinically plausible and meaningful risk factors were chosen to develop the risk scoring model. Five risk factors were significant in the final multivariable logistic model (Table 4): 20–24-year-old mothers, no income received from family members, unplanned pregnancy, no knowledge of when the last HIV test result was received, and no knowledge about the infant’s father’s HIV status. The validation data set was not consistent with the development data set; where only two risk factors were found to be significant: age of the mother and no knowledge of the infant’s father’s HIV status. The three models’ discriminatory ability was assessed to determine which provided the best predictive accuracy, based on the development and validation data sets (Table 5).

**Table 4:** Final multivariable logistic regression of young mother’s HIV infection using the development and validation data sets for Model Three from the 2010, 2011–2012, and 2012–2013 prevention of mother-to-child transmission surveys, South Africa

		**Development Data Set (*N* = 4676)**				**Validation Data Set (*N* = 2690)**			
**Risk factor**		**Coefficient**	**Odds Ratio (95% CI)**	***P*-value**	Score (coefficient*10)	**Coefficient**	**Odds Ratio (95% CI)**	***P*-value**	Score (coefficient*10)
Age of mother (years)	< 20	0	1	-	**0**	0	1	-	**0**
	20–24	0.98	2.67 (1.98–3.61)	< 0.001	**10**	0.85	2.34 (1.82–3.02)	< 0.001	**9**
Planned pregnancy	No	0.43	1.53 (1.15–2.03)	0.003	**4**				
	Yes	0	1	-	**0**				
Knowledge of weeks pregnant when received last HIV test result	No	0	1	-	**0**				
	Yes	0.92	2.52 (1.56–4.06)	< 0.001	**9**				
Knowledge of HIV status of child’s father	No	0.81	2.26 (1.76–2.89)	< 0.001	**8**	0.62	1.86 (1.50–2.32)	< 0.001	**6**
	Yes	0	1	-	**0**	0	1	-	**0**
Income from family	No	0.38	1.46 (1.04–2.06)	0.029	**4**				
	Yes	0	1	-	**0**				

*CI* Confidence interval

The *p*-value was set at *p*-value < .05

**Table 5:** Summary of the discriminatory ability of the three models from the 2010, 2011–2012, and 2012–2013 prevention of mother-to-child transmission surveys, South Africa

	**Development Data Set**		**Validation Data Set**	
	**Discriminatory ability of risk model**	**Overall discriminatory ability based on 5 random samples**	**Discriminatory ability of risk model**	**Overall discriminatory ability based on 5 random samples**
	**AUROC** **(95% CI)**	**Mean AUROC**	**AUROC** **(95% CI)**	**Mean AUROC**
Model I	0.73 (0.70–0.76)	0.72	0.69 (0.65–0.73)	0.71
Model II	0.71 (0.68–0.74)	0.70	0.72 (0.68–0.75)	0.70
Model III	0.69 (0.68–0.71)	0.66	0.63 (0.60–0.65)	0.67

*CI* Confidence interval

Model I had the best discriminatory ability, with the highest AUROC for both the development (0.73, 95% confidence intervals (CI): 0.70–0.76) and validation (0.69, CI: 0.65–0.73) data sets. Model II had slightly lower AUROC for the development data set with 0.71 (CI: 0.68, 0.74) and an improved AUROC for the validation data set with 0.72 (CI: 0.68–0.75). Model III had the least discriminatory ability with an AUROC of 0.69 (CI: 0.68–0.71) for the development data set and a much lower AUROC of 0.63 (CI: 0.60–0.65) for the validation data set. The risk scoring models were re-analyzed five times using five randomly selected development and validation data sets. The development and validation data sets had consistent AUROC values across the three models, Model I and II showing reasonably acceptable AUROC of 0.72/0.71 and 0.70/0.70 respectively and Model III with poorer discriminatory ability (AUROC = 0.66/0.67).

### Odds ratios (OR) of HIV risk score

**Fig. 1:**
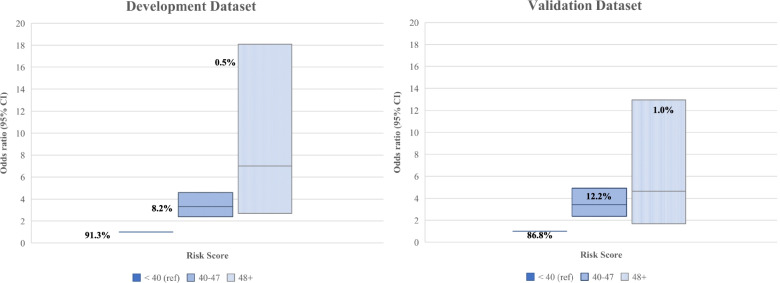
Odds ratio for risk scores of the development and validation datasets with percentages of women in each category

**Fig. 2:**
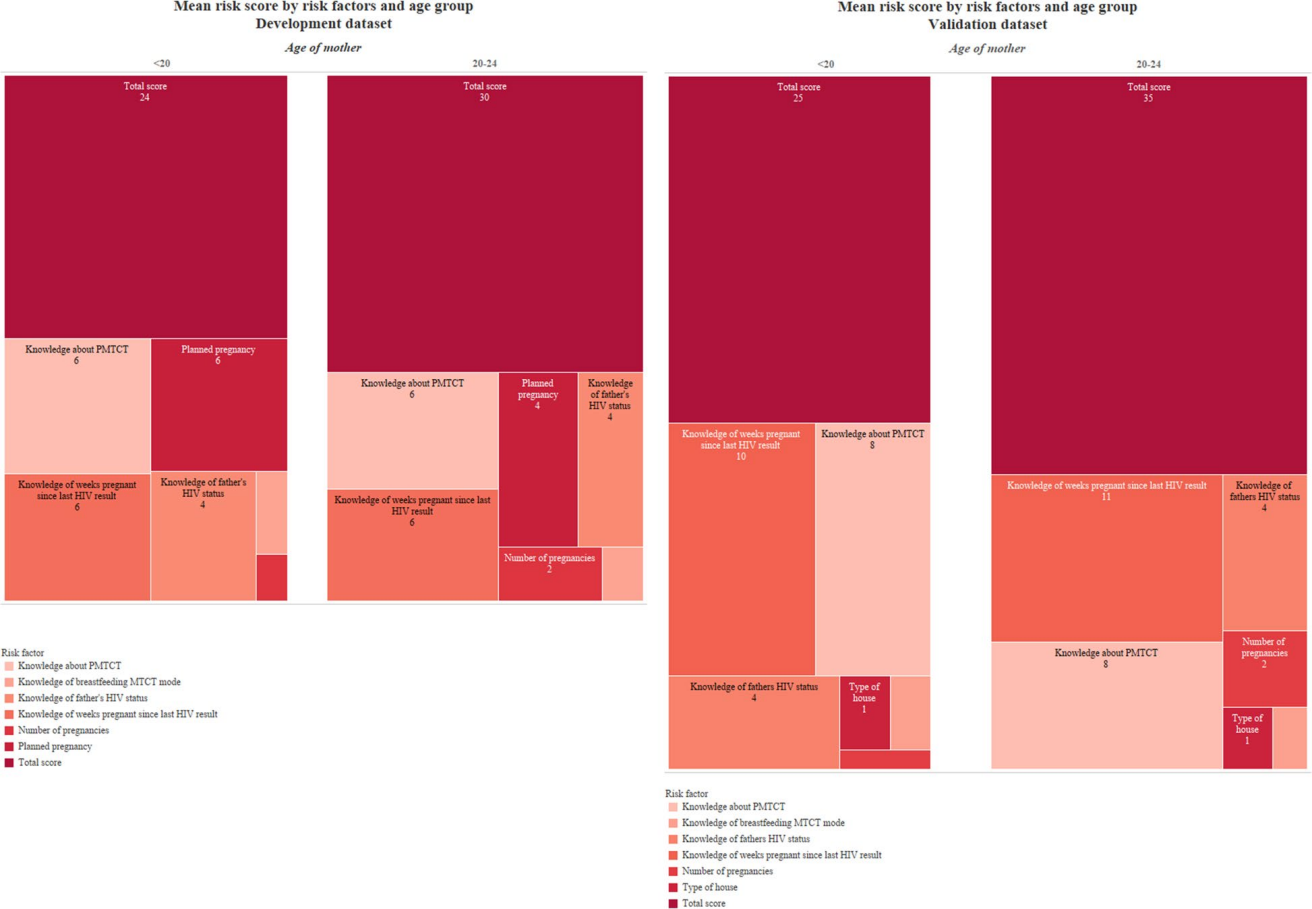
Mean risk scores of Model II stratified by age of mother, for the development and validations datasets

Subject-specific risk scores were calculated by adding the individual scores generated from Model II. The risk scores were then categorized into three groups (Fig. 1): low, moderate, and high risk. The odds ratios show an increasing linear trend towards higher risk of acquiring HIV with higher risk scores. Mothers with scores of 40–47 had 3.31 (CI: 2.39, 4.60) times greater odds of self-reported HIV infection than those with scores of less than 40, and those with scores greater than or equal to 48 had 7 (CI: 2.71, 18.09) times greater odds of acquiring HIV compared with mothers with scores of less than 40. The odds ratios were relatively similar between the development and validation data sets for mothers with scores between 40–47 but differed for mothers with scores greater than or equal to 48, where the validation data set showed that mothers were 5 (CI: 1.68, 12.95) times more likely to acquire HIV compared to those with scores of less than 40. Figure 2 shows the mean total risk scores for young women less than 20 years and between 20–24 years. The mean risk score was higher for mothers aged 20–24 years, in both the development and validation data sets. Knowledge about the PMTCT programme, knowing the HIV test results and not having a planned pregnancy contributed the most in those mother’s risks. Mothers younger than 20 years old had a mean risk score of 24 in the development data set. Similar risk factors seemed to contribute for mothers younger than 20 years, in addition to knowledge about the father’s HIV status.

### Performance of risk score

Additional analyses were done to evaluate the performance of the risk prediction model and determine the best cut-point of identifying those at highest risk, by assessing different cut-off points for Model II. The AUROC for the development data set was 0.71 (Table 6), and for the validation data set was 0.72 which means that the model has an approximate 72% chance of distinguishing young women’s risk of self-reporting HIV infection.

**Table 6:** Performance of risk score of Model II based on the development dataset from the 2010, 2011–2012, and 2012–2013 prevention of mother-to-child transmission surveys, South Africa. (*n* = 4222, AUROC = 0.71)

Cut point	Sensitivity	Specificity	Non-cumulative proportion of women at each cut point (%)
(> = 0)	100.00%	0.00%	0.02
(> = 7)	100.00%	0.03%	0.69
(> = 8)	99.21%	0.71%	0.21
(> = 11)	99.21%	0.93%	0.21
(> = 12)	99.21%	1.16%	0.09
(> = 14)	99.21%	1.26%	2.91
(> = 15)	98.82%	4.33%	3.86
(> = 16)	98.03%	8.39%	0.4
(> = 18)	98.03%	8.82%	1.42
(> = 19)	96.85%	10.26%	1.23
(> = 20)	96.06%	11.52%	0.07
(> = 21)	96.06%	11.59%	2.3
(> = 22)	96.06%	14.04%	16.84
(> = 23)	90.16%	31.58%	3.77
(> = 24)	89.76%	35.56%	0.02
(> = 25)	89.76%	35.58%	1.47
(> = 26)	88.19%	37.05%	6.8
(> = 27)	84.25%	44.03%	1.33
(> = 29)	83.46%	45.39%	10.61
(> = 30)	76.38%	56.22%	14.09
(> = 31)	60.24%	70.16%	0.43
(> = 33)	59.84%	70.59%	7.32
(> = 34)	49.21%	77.70%	5.85
(> = 35)	40.94%	83.39%	0.09
(> = 37)	40.94%	83.49%	8
(> = 38)	25.59%	91.03%	1.28
(> = 41)	22.83%	92.21%	7.15
(> = 42)	5.91%	98.74%	0.26
(> = 45)	5.12%	98.97%	0.76
(> = 49)	2.36%	99.60%	0.52
(> 49)	0.00%	100.00%	

The development dataset had a high sensitivity for cut-off values ≥ 23 (greater than 90%) and low specificity at 32% i.e., false positives would be high and PrEP use could be wasteful. For a cut-off point of ≥ 27 the sensitivity is 84% yielding 16% false negatives, and a specificity of 44%. The validation data set showed that for cut-off values of ≥ 31, the sensitivity and specificity was 86% and 45% respectively, yielding an acceptable balance of false negatives (14%) and false positives (55%) (Table 7).

**Table 7:** Performance of risk score of Model II based on the validation dataset from the 2010, 2011–2012, and 2012–2013 prevention of mother-to-child transmission surveys, South Africa. (*n* = 2110, AUROC = 0.72)

Cut point	Sensitivity	Specificity	Non-cumulative proportion of women at each cut point (%)
(> = 0)	100.00%	0.00%	0.28
(> = 4)	100.00%	0.26%	0.09
(> = 8)	100.00%	0.36%	0.38
(> = 9)	100.00%	0.77%	0.09
(> = 10)	100.00%	0.88%	0.57
(> = 12)	100.00%	1.49%	1.75
(> = 13)	98.81%	3.30%	0.28
(> = 14)	98.81%	3.61%	0.28
(> = 16)	98.81%	3.92%	1.00
(> = 17)	98.21%	4.95%	0.24
(> = 18)	98.21%	5.20%	1.61
(> = 19)	98.21%	6.96%	1.18
(> = 20)	98.21%	8.24%	1.80
(> = 21)	97.62%	10.15%	3.03
(> = 22)	97.62%	13.45%	8.20
(> = 23)	95.24%	22.15%	0.81
(> = 24)	95.24%	23.03%	0.95
(> = 25)	94.64%	24.01%	2.56
(> = 26)	94.05%	26.74%	3.27
(> = 27)	92.86%	30.19%	1.80
(> = 28)	91.07%	31.99%	0.33
(> = 29)	91.07%	32.35%	2.89
(> = 30)	90.48%	35.45%	9.19
(> = 31)	85.71%	45.03%	12.37
(> = 32)	77.98%	57.81%	0.19
(> = 33)	77.98%	58.01%	1.85
(> = 34)	76.19%	59.87%	3.46
(> = 35)	72.02%	63.27%	11.80
(> = 37)	53.57%	74.50%	0.71
(> = 38)	52.38%	75.17%	1.52
(> = 39)	50.00%	76.61%	12.27
(> = 41)	31.55%	88.36%	0.28
(> = 42)	29.76%	88.51%	0.28
(> = 43)	28.57%	88.72%	8.72
(> = 45)	9.52%	96.55%	0.09
(> = 47)	9.52%	96.65%	2.84
(> = 51)	2.98%	99.18%	0.71
(> = 55)	1.19%	99.79%	0.28
(> 55)	0.00%	100.00%	

## Discussion

In this study, we developed and internally validated a risk scoring tool to identify pregnant or lactating young women (≤ 24 years old) at high risk of HIV acquisition pre-pregnancy, antenatally and during breastfeeding, using data from three nationally representative South African PMTCT surveys. We presented three models using different methodologies to determine which risk factors could be combined to create a tool with high predictive accuracy, for easy use at the primary level of the health care system, to identify pregnant and lactating women at most risk of HIV infection. Findings from Model I of our study show that young maternal age (20–24 years), Black African ethnicity, informal house structure, having a flush toilet, two or more pregnancies, unplanned pregnancy, mothers who had knowledge of when they received their last HIV test result, no knowledge of the infant’s father’s HIV status, no knowledge of breastfeeding as a mode of MTCT, and knowledge of PMTCT programme were significant risk factors. In this model, the AUROC was 0.73 in the development dataset and 0.69 in the validation dataset suggesting that the model has a 70% chance of predicting the risk of HIV acquisition in young women. In Model II, ethnicity was removed, and significant risk factors were comparable to Model I except for toilet type and unplanned pregnancy. From the Model II development dataset, approximately 58% of the population had a risk score cut-off value of ≥ 27, with a sensitivity of 84% and a specificity of 44%. Thus, at this cut-off value, 44% of young pregnant and lactating women are determined to be high risk and would qualify for PrEP eligibility, allowing targeted implementation and intensified adherence support. In Model II the AUROC was 0.71 and 0.72 in the development and validation data sets respectively. We therefore recommend that in low resource, high HIV prevalence settings where PrEP availability, access and adherence may be limited, targeting PrEP and other HIV prevention strategies to young pregnant women with a cut-off score of ≥ 27 using Model II could maximise the impact of prevention strategies.

Risk factors such as young age, no knowledge of male partner’s HIV status and having multiple children identified in our risk scoring tool were also identified in other risk scoring tools among HIV uninfected non-pregnant women [10, 22]. The influence of Black African ethnicity (Model I) on an increased risk of HIV acquisition may be attributed to the high proportion of young Black pregnant/lactating women (90%) who were enrolled in the surveys compared to other ethnicities (10%). One of the reasons this finding is particularly relevant in South Africa is the Apartheid legacy which caused Black African females to be disproportionately affected by the HIV epidemic [12, 23, 24]. A population-based household survey in South Africa showed that the differences in HIV prevalence between the different ethnic groups was 20.6%, 5.8%, 1.3% and 0.9% among Black African, Coloured, Indian/Asian and White females respectively [12].

Our results show that young women who had an unplanned pregnancy have an increased risk of HIV acquisition. Unplanned pregnancy is higher amongst adolescents compared to adults and pregnant young women have an increased risk of MTCT and poorer maternal and infant health outcomes than older women [25, 26]. Findings by Floridia et al. show that younger women had a lower rate of planned pregnancy (OR 0.50, CI: 0.36–0.69) and were more frequently diagnosed with HIV in pregnancy (OR 3.29, CI: 2.54–4.25) [27].

Our findings also show that having knowledge of the PMTCT programme and lack of knowledge of breastfeeding as a mode of MTCT increases the risk of HIV acquisition during pregnancy and lactation. The former may relate to the self-identification of risk amongst young women at highest risk of HIV acquisition; thus, they deliberately found out about the PMTCT programme. Questions relating to knowledge of the PMTCT programme or modes of MTCT transmission in our study were limited, the level and intensity of knowledge, understanding, and source of information were not asked in our study. Qualitative findings from mothers attending a South African clinic reveal that although the majority of respondents in that study were aware of MTCT, how it could be prevented, and the importance of treatment adherence as a prevention measure, they lacked knowledge and understanding about the modes of transmission of MTCT [28]. In particular, most mothers did not understand the risk of MTCT after birth and failed to mention breastfeeding as a mode of transmission.

Identifying pregnant young women most at risk of HIV acquisition using a tool such as the one we propose will allow a cost effective and targeted approach to PrEP, rather than a universal approach, in areas where there are concerns about the safety of PrEP during pregnancy or where PrEP availability is limited. It would also allow for targeted support for these women in areas where the health system is stretched beyond capability. Young pregnant women may have a high risk on some factors and low risk on others, therefore a risk scoring tool comprising several risk factors calculates overall risk and allows for the tailored implementation of preventive interventions. The application of risk scoring tools has benefits at both clinical research (improving screening and selecting the most eligible participants) and at a primary health care level (improving cost effectiveness and use of resources by targeting those most at risk).

### Strengths and limitations

One of the key strengths of this analysis is the use of three nationally representative datasets which allows this risk scoring tool after validation to be generalizable to the general population. To our knowledge, this is only the risk scoring tool developed for pregnant and postpartum women in Southern Africa; the only other tool that we are aware of was developed in Kenya [5]. Moreover, this analysis focuses specifically on a risk scoring tool for young pregnant and lactating women (≤ 24 years).

Our study has some limitations which need to be considered when interpreting the results. Self-reported HIV status rather than incidence was used to develop the risk scoring algorithm. The data used is cross-sectional; longitudinal data would have been more suited to evaluate risk of HIV acquisition. Although the risk scoring tool in this study was developed using information from mothers attending immunisation services for their babies, who were screened and invited to participate, a limiting factor may have been that those most at risk of HIV acquisition may not have been included in the primary studies. Participants who received their care from mobile or private clinics were excluded from the study which may have influenced the type of participant enrolled in the study possibly skewing the data. Another limiting factor was the questions relating to knowledge of MTCT modes, PMTCT programme and antenatal care were limited and was based on maternal self-report. We also note that mothers’ knowledge of PMTCT and HIV transmission could have increased over time. However, literature from Bassey et al. who did a secondary analysis using data from the United Nation Children’s Fund Multiple Indicator Cluster Survey conducted in Nigeria in 2007, 2011, and 2016/2017 showed a significant decline in the knowledge of about MTCT among Nigerian women from 89.5% in 2007 to 80.3% and 78.2% in 2011 and 2016/17 respectively. Knowledge on the modes of MTCT for example breastfeeding declined from 87.6% in 2007 to 83.6 in 2016/17 respectively. [29] Darteh et al. using data from the 2016 South Africa Demographic and Health Survey showed that the prevalence of knowledge of mother to child transmission of HIV during pregnancy, delivery, breastfeeding was 87.0%, 81.1% and 80.3% respectively.[30] Pregnancy intention was measured using a single question rather than a validated measure for unplanned pregnancy. Some known behavioral and clinical factors for predicting HIV positivity, such as sexually transmitted infections, were not included in our models because the primary study did not evaluate them. The duration of recall was approximately one year for antenatal information.

### Future recommendations

Our risk scoring tool was limited to internal validation only, thus, there is a need for this risk scoring tool to be externally validated especially in public health settings to gain a better understanding of the applicability of the tool among young pregnant and lactating women in South Africa. In the absence of validation, we recommend that this risk scoring tool could be used:

- As a self-assessment risk tool (mobile application or online) for young women to understand their own risk and the potential benefits of prevention interventions such as PrEP.
- By healthcare providers to foster dialogue about user’s risk perception thus promoting a patient-centred approach by allowing users to make informed decisions about PrEP and other prevention interventions.

## Conclusion

In a vulnerable population such as young pregnant or lactating women, extra effort is required to minimise their risk of HIV acquisition. The application of our risk scoring tool can be used as a stepping stone to allow early identification of those pregnant/lactating women most at-risk of HIV acquisition in health systems with limited capacity or where PrEP access, availability, and adherence is limited. The scoring tool provides the opportunity to maximise provision of combination prevention interventions such as PrEP to this at-risk population thereby reducing new infections. Further validation using updated data and operational research are required to test the feasibility and performance of this risk scoring tool at primary health care level.

## Data Availability

The datasets used and analysed during the current study are available from the senior author (Ameena Goga: Ameena.Goga@mrc.ac.za) on reasonable request.
